# Multiple regulatory roles of the transfer RNA-derived small RNAs in cancers

**DOI:** 10.1016/j.gendis.2023.02.053

**Published:** 2023-04-13

**Authors:** Yu Zhang, Xinliang Gu, Yang Li, Yuejiao Huang, Shaoqing Ju

**Affiliations:** a Medical School of Nantong University, Nantong University, Nantong, Jiangsu 226001, China; b Department of Laboratory Medicine, Affiliated Hospital of Nantong University, Nantong, Jiangsu 226001, China; c Research Center of Clinical Medicine, Affiliated Hospital of Nantong University, Nantong, Jiangsu 226001, China; d Department of Medical Oncology, Affiliated Hospital of Nantong University, Nantong, Jiangsu 226001, China

**Keywords:** Biogenesis, Biological function, Cancer, Mechanism, Therapeutic strategy, tsRNAs

## Abstract

With the development of sequencing technology, transfer RNA (tRNA)-derived small RNAs (tsRNAs) have received extensive attention as a new type of small noncoding RNAs. Based on the differences in the cleavage sites of nucleases on tRNAs, tsRNAs can be divided into two categories, tRNA halves (tiRNAs) and tRNA-derived fragments (tRFs), each with specific subcellular localizations. Additionally, the biogenesis of tsRNAs is tissue-specific and can be regulated by tRNA modifications. In this review, we first elaborated on the classification and biogenesis of tsRNAs. After summarizing the latest mechanisms of tsRNAs, including transcriptional gene silencing, post-transcriptional gene silencing, nascent RNA silencing, translation regulation, rRNA regulation, and reverse transcription regulation, we explored the representative biological functions of tsRNAs in tumors. Furthermore, this review summarized the clinical value of tsRNAs in cancers, thus providing theoretical support for their potential as novel biomarkers and therapeutic targets.

## Introduction

The mechanisms of tumorigenesis and progression have been the focus of research. Among them, noncoding RNAs (ncRNAs), a class of RNAs that cannot be coded as proteins, are widely present in organisms and play a critical role in cancer progression.^1^^,^^2^ The development of next-generation sequencing (NGS) technology and bioinformatics analysis has led to the discovery of an emerging class of ncRNAs: transfer RNA-derived small RNAs (tsRNAs).

In the eukaryotic nucleus, RNA polymerase III (RNA Pol III) first transcribes tRNA genes into precursor tRNAs (pre-tRNAs).^3^ During the maturation of tRNAs, the 5′-lead sequence and the 3′ polyuracil (poly-U) tail of pre-tRNAs are cleaved by ribonuclease P (RNase P) and endonuclease Z (RNase Z)/cytoplasmic homologous ribonuclease Z2 (ELAC2), respectively. Subsequently, the “CCA” sequence is added to the 3′ end by nucleotidyl transferase, and then pre-tRNAs fold into the cloverleaf secondary structures of mature tRNAs by post-transcriptional modification.^4^, ^5^, ^6^, ^7^

Extensive evidence indicates that tsRNAs are generated from transcripts of tRNAs cleaved by nucleases in response to stress and have unique biological functions.^8^^,^^9^ In this review, we presented the classification and biogenesis of tsRNAs and summarized the latest action mechanisms of tsRNAs. In addition, we summarized the representative biological functions of tsRNAs in tumors and reported their advances as novel biomarkers and therapeutic targets in cancer.

## Classification of tsRNAs and corresponding biogenesis

**Figure 1:**
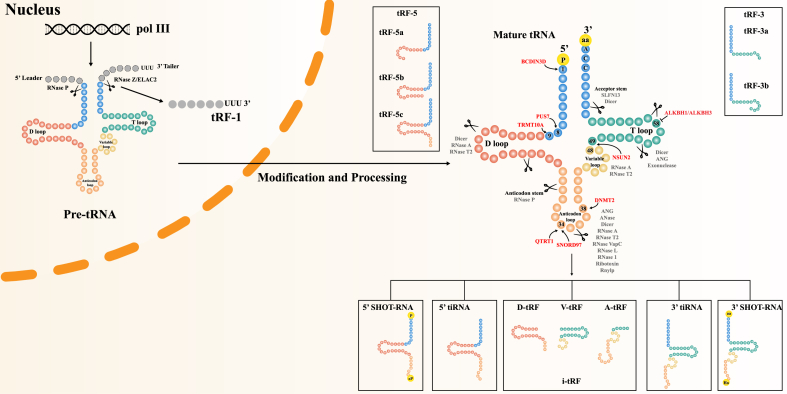
Classification and biogenesis of tsRNA. tsRNAs can be divided into two categories: tRNA halves (tiRNAs) and tRNA-derived fragments (tRFs). 5′ tiRNA contains the 5′ end of mature tRNA to the end of the anticodon loop, while 3′ tiRNA extends from the anticodon loop to the 3′ end of mature tRNA. The 5′ SHOT-RNAs are characterized by a phosphate at the 5′ end and a 2′,3′-cyclic phosphate at the 3′ end, whereas the 3′ SHOT-RNAs have a hydroxyl group at the 5′ end and an amino acid at the 3′ end. tRFs can be divided into tRF-1, tRF-3, tRF-5, and i-tRF. During the maturation of the 3′-terminus of tRNA, tRF-1 is released by cleavage of RNase Z/ELAC2. tRF-3 has two subtypes: tRF-3a and tRF-3b. tRF-5 can be classified into tRF-5a, tRF-5b, and tRF-5c. i-tRF is the internal region of mature tRNA, including A-tRF, V-tRF, and D-tRF. The biogenesis of tsRNAs is regulated by tRNA modification.

Based on the differences in the cleavage sites of nucleases on mature tRNAs or pre-tRNAs, tsRNAs can be divided into two categories: tRNA halves (tiRNAs) and tRNA-derived fragments (tRFs) (Fig. 1).

### tRNA halves (tiRNAs)

Under stressful conditions such as oxidative stress, heat shock, UV radiation, hypoxia, and viral infection, the middle site of the anticodon loop of mature tRNAs will be cleaved by nucleases to generate tiRNAs.^10^ In addition, tiRNAs are also present under non-stress conditions. They can be detected in body fluids such as serum/plasma, sperm, saliva, and urine, as well as in extracellular vesicles (EVs).^11^, ^12^, ^13^, ^14^, ^15^, ^16^

tiRNAs can be subclassified into 5′ tiRNA and 3′ tiRNA. 5′ tiRNA contains the 5′ end of mature tRNA to the end of the anticodon loop, while 3′ tiRNA extends from the anticodon loop to the 3′ end of mature tRNAs.^10^^,^^17^ The length of 5′ tiRNA is approximately 31–36 nucleotides (nt), slightly shorter than that of 36–41 nt of 3′ tiRNA.^18^ Another type of tsRNAs, called sex hormone-dependent tRNA-derived RNAs (SHOT-RNAs), are produced by sex hormone-induced angiogenin (ANG, known as RNY1 in yeast) cleavage of fully acylated mature tRNAs and are abundantly expressed in breast cancer (BC) and prostate cancer (PC). The 5′ SHOT-RNAs are characterized by a phosphate at the 5′ end and a 2′,3′-cyclic phosphate at the 3′ end, whereas the 3′ SHOT-RNAs have a hydroxyl group at the 5′ end and an amino acid at the 3′ end.

tiRNAs are mainly produced by the cleavage of ANG, a member of the vertebrate-specific secreted ribonuclease a (RNase A) superfamily. Under normal conditions, ANG in the nucleus is inactivated by binding to ribonuclease/angiogenin inhibitor 1 (RNH1), which is sensitive to oxidative stress. While under stress, ANG will be transferred to the cytoplasm to separate from RNH1 and then be activated to cleave mature tRNAs to produce tiRNAs.^19^, ^20^, ^21^ However, researchers investigating ANG-mediated tsRNAs discovered that only a fraction of tsRNAs changed their expression when ANG was knocked out, pointing to the involvement of other endonucleases.^22^ It has been shown that the RNase T2 family has a significant role in producing tiRNAs in *Saccharomyces cerevisiae*, *Arabidopsis*, and *Tetrahymena*.^23^, ^24^, ^25^ Additional nucleases involved in the processing of tiRNAs are RNase L, endonuclease V, RNase I, and Schlafen13/SFLN13.^26^, ^27^, ^28^, ^29^

### tRNA-derived fragments (tRFs)

tRFs are fragments generated by the nuclease cleavage of tRNAs and have a length of 14–30 nt, similar to miRNAs.^30^, ^31^, ^32^, ^33^ Depending on the cleavage sites, tRFs can be divided into tRF-1, tRF-3, tRF-5, and i-tRF.^34^ During the maturation of the 3′-terminus of tRNA, tRF-1 is released by cleavage of RNase Z/ELAC2 with a length of 14–30 nt. Because the 3′ end of tRF-1 contains a poly U sequence, it is also called 3′ U-tRF.^18^^,^^34^^,^^35^ tRF-3 is produced by cleavage of the TΨC loop of mature tRNA, so its tail contains a CCA structure. Based on its cutting position (U/A or U/U), tRF-3 can be divided into tRF-3a (18 nt) and tRF-3b (22 nt).^18^^,^^36^ tRF-5 contains the 5′ end of tRNA and is generated by cleavage of the D-loop or the arm stem between the anticodon loop and D-loop. tRF-5 can be classified into three subclasses: tRF-5a (14–16 nt), tRF-5b (22–24 nt), and tRF-5c (28–30 nt).^18^^,^^34^ The fourth tRF is i-tRF (previously named tRF-2), which originates primarily from the internal region of mature tRNA.^37^ Regarding i-tRFs, A-tRFs are formed by cleavage of the anticodon loop, V-tRFs by variable region cleavage, and D-tRFs by cleavage of the D-stem.^38^

In general, tRFs may be generated through Dicer-dependent and independent pathways. For example, Cole et al confirmed that the biogenesis of tRFs derived from tRNA^Gln^ depends on Dicer *in vivo*, and inhibition of Dicer significantly reduces the corresponding tRFs.^39^ Similar phenomena were observed in Dicer-knockout mutants of mature B cells and Arabidopsis.^40^^,^^41^ However, researchers have found evidence that tRFs are generated via a Dicer-independent pathway in *Arabidopsis* and other eukaryotes.^42^, ^43^, ^44^, ^45^ In particular, tRF-3-mediated gene repression is not impaired in Dicer-knockout cells and showed an improvement,^46^ suggesting that Dicer may be replaced by other tRNA processing components. For example, RNase *Z* can cleave tRNA to produce tRF-1,^47,48^ and ANG may induce the production of tRFs.^49^ Furthermore, the *Arabidopsis* RNase T2 family gene RNS1 (Ribonuclease 1) is involved in the formation of tRF-5a/3 b.^42^ Other cutting enzymes have been summarized in recent reviews.^50^^,^^51^

### Novel tRFs

A novel set of tRFs derived from pre-tRNAs was reported to be associated with the loss of polyadenylation factor I subunit 1 CLP1 kinase activity.^52^ The human tRNA splicing endonuclease (TSEN) cleaves tRNA at the 3′ intron–exon junction. CLP1 is part of the TSEN complex and is capable of phosphorylating the 3′ exon of tRNA.^53^^,^^54^ In neurodegenerative diseases, the CLP1 (R140H) mutant fails to interact with TSEN, preventing the 5′ OH of the 3′ exons from being phosphorylated, thereby blocking the subsequent cleavage steps. As a result, tRNA introns or 3tiR-like fragments from the 3′ exons accumulated in the cells.^52^^,^^55^ Therefore, the actual categories of tsRNAs are more diverse than those identified so far, and further study is necessary.

### Impact of tRNAs modifications on the biogenesis of tsRNAs

Modifications on tRNAs can not only affect the function of tRNAs but also protect them from being cleaved by nucleases, so their absence may induce the production of tsRNAs (Fig. 1).^56^^,^^57^

Post-transcriptionally, methylation is a common chemical modification of tRNAs.^58^ DNMT2 in *Drosophila* has been reported to mediate 5-methylcytosine (m5C) methylation at C38 of tRNA-AspGTC, tRNA-ValAAC, and tRNA-GlyGCC, which could protect them from ANG-mediated cleavage.^59^^,^^60^ By contrast, deficiency of NSUN2 in the neurons of mice decreases the m^5^C_48/49_ modifications on tRNAs, resulting in increased expression of 5′ tiRNAs.^61^^,^^62^ Additionally, the deficiency of TRMT10A causes a decrease in methylation of guanosine at m^1^G_9_ of tRNAs, resulting in a significant increase of 5′-tRFs^Gln^.^63^ The methyltransferase BCDIN3D prompts the 5′-phosphate methylation of tRNA^His^ and thereby protects it from cleavage to tRF-3.^64^^,^^65^ In addition, ALKBH1 and ALKBH3 are demethylases of 1-methyladenosine (m^1^A) of tRNAs, causing the biogenesis of tsRNAs.^66^^,^^67^ Pseudouridylation synthase 7 (PUS7), which is enriched in human embryonic stem cells (hESCs), can modify uridine (U) to pseudouridine (ψ) at the U8 position of tRNAs thereby mediating the production of tRFs.^68^ Furthermore, the hypermodified 7-deaza-guanosine queuosine (Q) is found at tRNA position 34, the wobble anticodon. Q modification in human cells is catalyzed by QTRT1/QTRT2 enzymes, which protects cognate tRNA^His^ and tRNA^Asn^ from cleavage.^69^ Other tRNA modifications have been listed in Figure 1.

## Characteristics of tsRNAs in the organism

### The expression of tsRNAs is tissue-specific

tsRNAs are dynamically regulated by the organism during development. For example, there are specific tRFs derived from tRNA-Arg-TCT-4-1 expressed in the brain, while tRFs derived from tRNA-Arg-CCG-2-1 are found in the ovary, heart, and skeletal muscle.^70^ As in other species, tissue-specific enrichment of tRFs may be a common phenomenon.^11^^,^^47^ For example, the level of tRF Glu-CTC-5A in flowers of *Arabidopsis* is higher than that in seedlings,^71^ whereas phosphate-induced tRF Asp-5a is accumulated primarily in the roots and not in buds.^72^ Additionally, Alves et al observed that tRF-5 and tRF-3 are also enriched in specific tissues or developmental stages of *Arabidopsis*.^42^ This is also the case for the tRF accumulation in *Phytophthora infestans* and *Phytophthora sojae*.^43^^,^^73^

### Specific subcellular localization of tsRNAs

tsRNAs are mainly located in cells, with only a small fraction in the peripheral circulation.^74^ However, their initial location and distribution within the cells are different. Most tiRNAs are located in the cytoplasm due to the translocation of ANG from the nucleus to the cytoplasm to cleave mature tRNAs into tiRNAs.^75^ There are also a small number of tiRNAs in the nucleus and mitochondria.^11^^,^^30^^,^^61^ tRF-1 is mainly localized in the cytoplasm. The RNaseZ has been reported to be responsible for generating tRF-1 directly in the cytoplasmic pool.^47^^,^^48^^,^^76^ According to Liao et al, the 3′ end of tRNA is processed in the nucleus, but tRF-1001 is located in the cytoplasm,^35^ meaning that tRF-1 would first be released in the nucleus and then transported to the cytoplasm by a specific mechanism.^35^^,^^44^ Additionally, tRF-3 is primarily distributed in the cytoplasm.^30^^,^^44^ Despite being produced in the cytoplasm, tRF-5 is mainly located in the nucleus in a meta-analysis of small RNA data,^39^^,^^44^ suggesting that it may be transported to the nucleus via a similar transport mechanism.^32^^,^^77^^,^^78^

## In-depth exploration of the action mechanisms of tsRNAs

**Figure 2:**
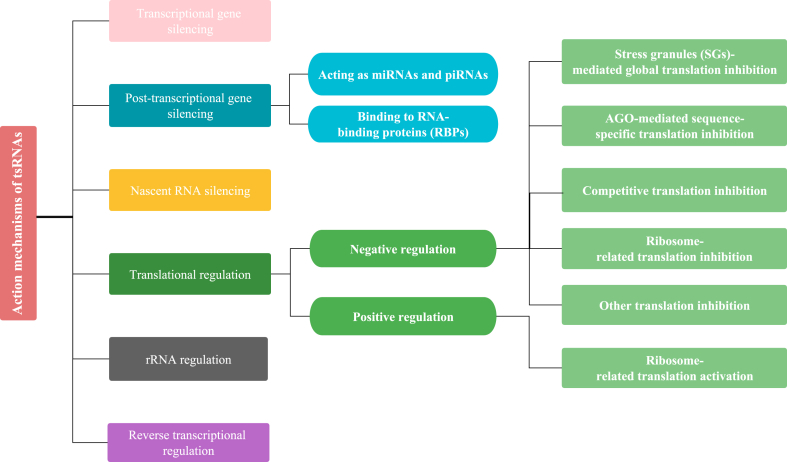
Flowchart of the action mechanisms of tsRNAs.

tsRNAs are small noncoding RNAs (sncRNAs) that can function as regulators through various mechanisms. Based on the latest research on tsRNAs, we summarized their mechanisms into the following six categories, including transcriptional gene silencing, post-transcriptional gene silencing, nascent RNA silencing, translational regulation, rRNA regulation, and reverse transcriptional regulation (Fig. 2).

### Transcriptional gene silencing (TGS)

**Figure 3:**
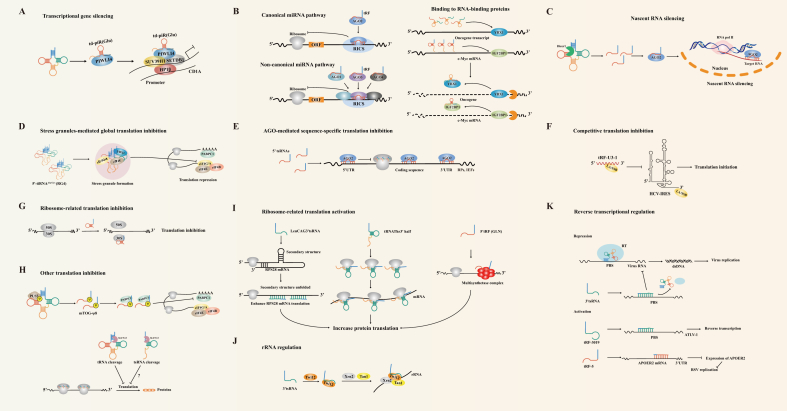
Action mechanisms of tsRNAs. **(A)** td-piR (Glu) interacts with PIWIL4 protein to inhibit transcription. **(B)** tsRNAs play canonical or non-canonical miRNAs-like roles in regulating gene expression by binding to AGO2 or other AGO proteins; tsRNAs serve as protein decoys to isolate RBPs from target RNAs. **(C)** tsRNAs direct AGO2 to target introns via nascent RNA silencing (NRS). **(D)** SGs were induced by the binding of 5′ tiRNAs to YBX1, resulting in the isolation of translation initiation factors. **(E)** tsRNAs in *Drosophila* preferentially inhibit the translation of RPs and IEFs through antisense pairing in an AGO2-dependent manner. **(F)** tRF-U3-1 competitively inhibits La/SSB-dependent viral protein translation. **(G)** Val-tRF binds to the 16 S rRNA of the 30 S ribosomal subunit, causing mRNA to be displaced from the initiation complex and thus weaken translation. **(H)** The binding of PUS7 to tRNAs induces the production of mTOG-ψ8, which preferentially binds to PABPC1, leading to the displacement of translation initiation factors thereby inhibiting translation; SLFN13 directly cleaves tRNAs and tsRNAs, leading to global translational repression. **(I)** LeuCAG3′tsRNA binds to the double-stranded target sites of RPS28 mRNA and unfolds a secondary hairpin structure, thereby enhancing translation; tRNAThr3′half binds to ribosomes and multimers and enhances protein synthesis by facilitating mRNA loading to actively translated ribosomes; 5′tRF Gln19 interacts with Human Multisynthetase Complex (MSC) to increase translation. **(J)** tsRNAs affect rRNA processing by recruiting Twi12, Xrn2, and Tan1. **(K)** tsRNAs participate in the regulation of viral reverse transcription by blocking or mimicking the action of tRNAs.

It has been reported that the P-element-induced wimpy testis (PIWI)/PIWI-interacting small RNAs (piRNAs) complex can recruit histone methyltransferase Su (var) to H3K9 to maintain chromatin inhibition after recruiting to the epigenetic factor HP1a.^79^ tsRNAs, similar to piRNAs in length (26–31 nt), are also associated with PIWI proteins and can exert transcriptional repression in a piRNAs-like manner.^80^^,^^81^ For example, the expression of td-piR (Glu) derived from tRNA-Glu is much higher in human monocytes than in dendritic cells. In monocytes, td-piR (Glu) does not interact with Argonaute (AGO) proteins but rather with PIWIL4 proteins to form a complex, which then recruits H3K9 methyltransferases (SETDB1 and SUV39H1) as well as heterochromatin protein 1β (HP1β) to the CD1A promoter region, leading to significant repression of H3K9 methylation and transcription of CD1A, which is a novel epigenetic mechanism for regulating CD1A expression (Fig. 3A). While during the differentiation of monocytes into DC, the down-regulation of td-piR (Glu) expression leads to a decrease in H3K9 modification, which converts the CD1A promoter region into an auto chromatic state, thereby being recognized by different transcription factors and enhancing transcription.^82^

### Post-transcriptional gene silencing (PTGS)

#### Acting as miRNAs and piRNAs

tsRNAs can be loaded into AGO proteins, essential components of the RNA-induced silencing complex (RISC) that represses gene expression through RNA interference.^83^ There is evidence that AGO2 is the only protein in the AGO family to cleave target genes. tsRNAs can regulate gene expression by binding to AGO2 or other AGO proteins to play canonical or non-canonical miRNAs-like roles (Fig. 3B).^44^^,^^48^^,^^84^

tRF-T11 in Chinese yew can interact with AGO2 to inhibit the growth of ovarian cancer by directly targeting the oncogene TRPA1 through the RNAi pathway.^85^ tRF-3s can also play canonical miRNAs-like roles in regulating endogenous viral expression by binding to AGO2.^45^ However, Kumar et al found that tRF-5s and tRF-3s were associated with AGO 1, 3, and 4 but not with AGO2 in human HEK293 cells, suggesting that tRFs may play a role in RNA silencing through a non-canonical pathway.^44^ Furthermore, when Guan et al characterized tRFs loaded on AGO1 protein, they found that tRFs interact with target genes at the 3ʹUTR through a miRNAs-like manner.^86^ CU1276 in mature B cells can inhibit the expression of RPA1 mRNA by binding to its 3′UTR, thereby suppressing the proliferation of lymphoma cells and regulating the molecular response to DNA damage.^40^ Huang et al reported that tRF/miR-1280 inhibits the Notch signaling pathway by directly targeting the 3′UTR of Notch ligand JAG2 mRNA, thereby reducing tumor progression.^87^

It has been demonstrated that tsRNAs can act as piRNAs and play PTGS roles in human somatic cells,^88^ *T. thermophila*,^31^^,^^89^ and *B. mori*.^90^ ts-101 and ts-53 are found in both AGO1 and AGO2 complexes as well as Piwi-L2 complexes in chronic lymphocytic leukemia (CLL), suggesting that they may play similar roles to piRNAs.^91^ ts-53 has also been reported to inhibit the development of CLL by targeting the 3′UTR of TCL1.^91^ The above studies have shown that tsRNAs can target mRNAs like miRNAs and piRNAs, thereby regulating the post-transcriptional silencing of genes.

#### Binding to RNA-binding proteins (RBPs)

Impaired RBPs have been shown to play a role in various diseases.^61^^,^^92^^,^^93^ RBPs stabilize target RNAs by binding to them, while tsRNAs can serve as protein baits for isolating RBPs from RNAs and regulating gene expression (Fig. 3B).^94^^,^^95^ The classic Y box binding protein 1 (YBX1) is a multifunctional RBP, which can maintain RNA stability and promote tumor development by binding to oncogenic transcripts.^96^, ^97^, ^98^ Specific tRFs compete with the 3′UTRs of oncogene transcripts to bind to YBX1 in BC. As a result, YBX1 dissociates from oncogene mRNA and inhibits the proliferation of BC cells.^99^ Weidensdorfer et al selected tsGlnCTG in differentiated mouse embryonic stem cells (mESC) for proteomics analysis and found that it preferentially interacted with Igf2bp1.^100^ Igf2bp1 is a carcinogenic RBP involved in the maintenance of stem cells. It can prevent the intranuclear cleavage of c-Myc by binding to the coding region instability determinant (CRD) in the c-Myc coding sequence.^101^ The up-regulation of 5′-tsRNAs results in the isolation of Igf2bp1 from the complex, leading to endonucleolytic cleavage of c-Myc mRNA and thereby reducing its expression, ultimately promoting the transformation of mESC from a pluripotent state to a differentiated state.^100^

### Nascent RNA silencing (NRS)

Di et al proposed a novel gene silencing mechanism different from TGS and PTGS, which occurs in the nucleus and does not affect the transcriptional state of the gene.^102^ In this mechanism, Dicer1-dependent tsRNAs guide AGO2 to target introns through NRS in the nucleus, leading to the down-regulation of target genes (Fig. 3C). This is the first time that the nuclear function of RISC is applied to tsRNAs,^103^ which expands the vision of researchers on gene regulation. In large part, the single-stranded nature of tsRNAs explains their ability to participate in the NRS. According to David et al, small single-stranded RNA can bind to nuclear AGO2. In contrast, double-stranded siRNA can only bind to cytoplasmic AGO2,^104^ which may be due to the lack of auxiliary double-stranded RNA loading factors in the nucleus. Additionally, tsRNAs modification may facilitate their entry into the nucleus.^105^ It is also important to recognize that the biological significance of NRS is derived from its regulation of target genes implicated in a variety of diseases, which still needs to be elucidated further.^102^^,^^105^

### Translational regulation

#### Negative regulation

##### Stress granules (SGs)-mediated global translation inhibition

When the organism is exposed to adverse conditions, multiple stress responses can occur, and eukaryotic cells tend to conservatively synthesize metabolic energy to repair molecular damage. In this situation, SGs are formed in the cytoplasm, which helps reprogram translation and alleviates the depletion of energy within the cell.^106^ SGs are generally generated through two pathways, one of which relies on eukaryotic translation initiation factor (eIF)2α phosphorylation. After eIF2α is phosphorylated, the eIF2-GTP-tRNAiMet ternary complex is depleted, accompanied by the formation of SGs in the cytoplasm, which ultimately prevents translation initiation.^107^, ^108^, ^109^, ^110^ The other is the eIF2a phosphorylation independent pathway,^111^ in which SGs are mainly triggered by two compounds. The first class of compounds includes tiRNAs, sodium selenite, and hydrogen peroxide, which mainly interfere with the interaction of eIF4E and eIF4G. The other compounds include 15-deoxy-Δ(12,14)-prostaglandin J2, silvestrol, and pateamine A, primarily inhibiting the function of the RNA helicase eIF4A.^112^

It is worth noting that tiRNAs can inhibit translation by inducing the formation of SGs through an eIF2a phosphorylation-independent pathway.^21^ To be specific, 5′-tiRNA^Ala^ and 5′-tiRNA^Cys^ have a terminal oligonucleotide-G motif (TOG), and the TOG motif can promote the assembly of intermolecular RNA G-quadruplex (RG4). RG4 then replaces eIF4G by interacting with the HEAT1 domain in eIF4G, thereby inhibiting translation initiation.^113^, ^114^, ^115^ Furthermore, binding of 5′ tiRNAs to the shock domain of YBX1 induces the assembly of SGs, thereby isolating translation initiation factors and suppressing global translation (Fig. 3D). YBX1 knockouts, however, cannot fully reverse translation repression, indicating that this is not necessary for 5′ tiRNA-mediated translation inhibition.^115^, ^116^, ^117^

##### AGO-mediated sequence-specific translation inhibition

tsRNAs are conserved and ubiquitous in *Drosophila*. Through mRNAs sequencing and ribosome analysis of S2 cells transfected with single-stranded tsRNA mimics and mocks, the 5′ ends of tsRNAs can be antisense paired to 7-mer target sites at different positions of mRNA to regulate the expression of target genes. Luo et al found that ribosomal proteins (RPs) and translational initiation or elongation factors (IEFs) have a high target density of AGO2-bound tsRNAs (Fig. 3E). Under serum starvation conditions, AGO2 knockout experiments in S2 cells confirmed the dependence of tsRNAs-mediated translation inhibition on AGO2.^118^ Therefore, tsRNAs in *Drosophila* can preferentially inhibit the translation of RPs, IEFs, and other critical components of translation through antisense pairing in an AGO2-dependent manner, ultimately inhibiting the global translation.

##### Competitive translation inhibition

The cytoplasmic protein La/SSB can act as a molecular chaperone to bind to stem-loop IV of the HCV internal ribosome entry site (IRES), facilitating IRES-mediated translation initiation.^119^ However, under stress conditions, La/SSB is also able to bind and stabilize tRF-U3-1 in the cytoplasm, which results in a relatively reduced binding of La/SSB to IRES, thereby inhibiting IRES-mediated translation and exerting antiviral activity against HCV (Fig. 3F).^120^ Furthermore, the inhibitory effect of tRF-U3-1 on HCV IRES is lost in the absence of La/SSB.^120^ Thus, tRF-U3-1 can competitively inhibit La/SSB-dependent viral protein translation.

##### Ribosome-related translation inhibition

Gebetsberger et al found that Val-tRF in *Haloferax volcanii* binds to the 16 S ribosomal RNA (rRNA) of the 30 S ribosomal subunit in the absence of other cofactors, causing mRNAs to be displaced from the initiation complex and hence attenuating translation (Fig. 3G). Eukaryotic and bacterial systems also exhibit this effect, suggesting that this mechanism is conserved.^121^ Additionally, tsRNAs in yeast may inhibit translation by regulating tRNA aminoacylation through interactions with ribosome-associated aminoacyl-tRNA synthetase (AARS). However, the exact mechanism remains unknown.^122^

##### Other translation inhibition

As previously mentioned, PUS7 induces the production of specific tRF-5s upon binding to tRNAs. Since these tRF-5s contain a common oligoguanine motif at the 5′ ends, they are also named mTOG-ψ8. Under normal conditions, cytoplasmic poly(A)-binding protein-1 (PABPC1) interacts with translation initiation factors (eIF-4A/G and E) to form a closed-loop translation complex. Whereas under stress, mTOG-ψ8 preferentially binds to PABPC1, resulting in the displacement of eIF-4A/G and E from mRNAs, thereby inhibiting translation, but mTOG-U8 does not have this effect (Fig. 3H). The deletion of PUS7 and mTOG-ψ8 impairs early embryogenesis and affects hematopoiesis.^68^^,^^123^ Consequently, tsRNAs can regulate translation through similar self-modification. It has been reported that the Schlafen III (SLNF III) family protein SLFN13 is a eukaryotic RNase that cleaves cytoplasmic tRNAs and rRNAs in a site-specific and sequence-independent manner, resulting in global translation inhibition and effective anti-HIV activity.^29^ This mechanism may also apply to tsRNAs produced by SLFN13 cleavage (Fig. 3H).

#### Positive regulation

##### Ribosome-related translation activation

Ribosomal protein S28 (RPS28) is required for ribosomal 18 S rRNA biogenesis and is a component of the 40 S ribosomal subunit.^124^ RPS28 mRNA contains two targets that bind to LeuCAG3′tsRNA, one of which is located in the coding sequence (CDS) and can form a double strand with itself. Another target is located in the 3′UTR of RPS28 mRNA, which can form a secondary structure with the region containing the translation initiation site.^125^ It was reported that LeuCAG3′tsRNA unfolds a secondary hairpin structure after binding to the double-stranded target sites of RPS28 mRNA, enhancing translation in hepatoma cells (Fig. 3I).^126^ Fricker et al found that tRNAThr3′half is accumulated in *Trypanosoma brucei* in the presence of nutrient deficiency. During the recovery phase of starvation, tRNAThr3′half binds to ribosomes and multimers and enhances protein synthesis by facilitating mRNA loading to actively translated ribosomes (Fig. 3I).^127^ In addition, through stable isotope labeling of amino acids in cell culture (SILAC), immunoprecipitation, and mass spectrometry analysis, Keam et al found that a 19 nt 5′tRF Gln19 can interact with Human Multisynthetase Complex (MSC) to increase the translation of ribosome and poly (A)-binding proteins, but the specific mechanism needs further study (Fig. 3I).^128^

### rRNA regulation

In *Tetrahymena thermophila*, Couvillion et al observed that the Twi12 protein can bind fragments from the 3′ ends of mature tRNAs and subsequently recruit Tan1 protein and Xrn2 to form a pre-rRNA splicing complex, in which Xrn2 also has exonuclease activity. This complex regulates the synthesis of mature rRNAs by cleaving and processing precursor rRNAs (Fig. 3J).^129^ However, whether tRFs can enhance the synthesis process of rRNAs in other organisms needs further exploration.

### Reverse transcriptional regulation

Viruses can use the 3′ ends and TψC arms of specific tRNA as reverse transcription primers to interact with the primer binding sites (PBS) of viral single-stranded RNAs, which are then converted into double-stranded DNA by viral reverse transcriptase (RT) and integrated into the host genome to promote viral replication.^130^ However, Schorn et al reported that tRF-3s can compete with tRNAs for binding to PBS of endogenous retroviruses (ERVs), resulting in RT blocking, and thereby hindering viral reverse transcription (Fig. 3K).^131^ In HIV-infected T cells, the blocking effect of tsRNAs may also occur at the PBS site that hybridizes with the 3′ end of tRNA^Lys^, thereby reducing the copy of HIV-1 RNA.^132^ Thus, the blocking effect of tsRNAs may negatively affect the reverse transcription that requires host tRNAs as primers.

tsRNAs can also be used as primers for reverse transcription (Fig. 3K). For example, HTLV-1-infected cells can release virus particles containing tRF-3019. tRF-3019 is complementary to the PBS of HTLV-1, which can initiate HTLV-1 reverse transcription and increase viral amplification.^133^ Analogously, another study found that tRF5-Gly (CCC) and tRF5-Lys (CTT) promote viral replication after respiratory syncytial virus (RSV) infection.^134^ In addition, RSV infection can induce the production of tRF5-GluCTC, which inhibits the expression of apolipoprotein E receptor 2 (APOER2) by recognizing the target site of APOER2 (Fig. 3K).^135^

## The classical function of tsRNAs in cancers

In this section, we described the classical biological function of tsRNAs in cancers, including cell proliferation, tumor metastasis, cell apoptosis, metabolic disorders, and therapeutic resistance.

### Cell proliferation

**Figure 4:**
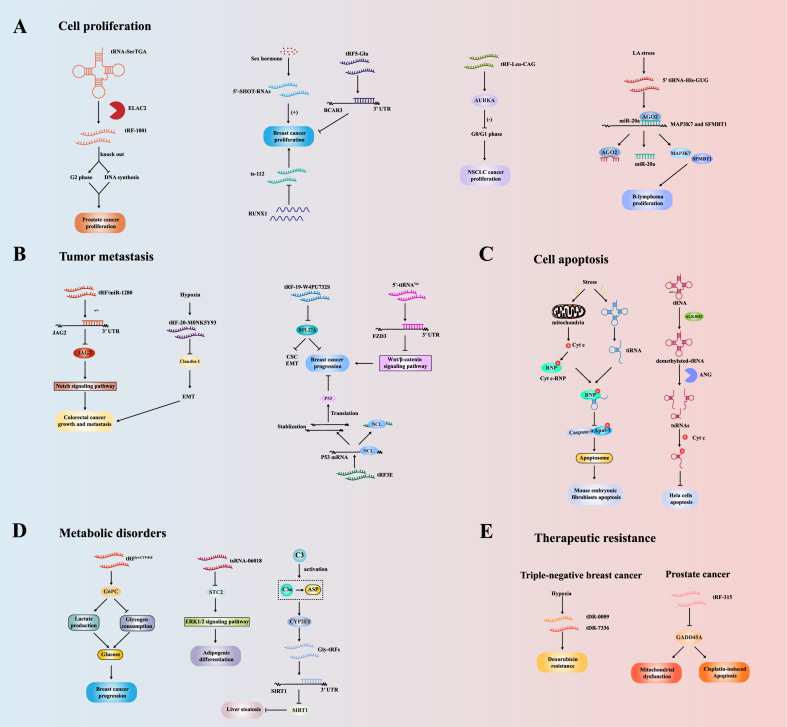
Biological functions of tsRNAs in cancers. **(A)** Knockout of tRF-1001 leads to reduced DNA synthesis and cell cycle arrest in the G2 phase, thereby inhibiting cell proliferation; 5′-SHOT-RNAs can promote BC proliferation; tRF5-Glu inhibits the proliferation by binding to the 3′UTR of BCAR3 mRNA; RUNX1 protects mammary epithelial cells from proliferation by inhibiting ts-112; tRF-Leu-CAG regulates the cell cycle by affecting AURKA activity; Lactate (LA)-induced 5′tiRNA-His-GUG competitively binds to AGO2, resulting in a decrease in the association of AGO2 with miR-20a thereby leading to an increase in the expression of target genes SFMBT1 and MAP3K7, and ultimately promoting the proliferation of B lymphoma cells. **(B)** tRF/miR-1280 inhibits the Notch signaling pathway by targeting the 3′UTR of JAG2, ultimately inhibiting CRC metastasis; tRF-20-M0NK5Y93 inhibits migration and invasion by targeting EMT-associated Claudin-1; tRF-19-W4PU732S promotes the progression of BC cells by inhibiting ribosomal protein-L27A (RPL27A), and can also induce EMT and cancer stem-like cells (CSC) phenotypes; 5′-tiRNA^Val^ inhibits the metastasis of BC cells by targeting FZD3 to inhibit the Wnt/β-catenin signaling pathway; tRF3E forms a complex with NCL, thereby disrupting the inhibitory effect of NCL on p53 mRNA translation, and thus inhibiting tumor growth. **(C)** tiRNAs interact with Cyt c and prevent it from binding to Apaf-1, hence inhibiting apoptosis; ALKBH3 can catalyze the demethylation in tRNAs, resulting in the generation of tsRNAs, which subsequently interact with Cyt c to prevent apoptosis. **(D)** tRF^Lys−CTT−010^ promotes TNBC progression by targeting the G6PC subunit to regulate lactate production and glycogen consumption; tsRNA-06018 regulates adipogenic differentiation in hMSCs by targeting STC2 through the ERK1/2 signaling pathway; C3 activation products C3a and Asp increase the expression of Gly-tRFs by mediating the expression of CYP2E1, and then Gly-tRFs inhibits liver steatosis by targeting the 3′UTR of SIRT1. **(E)** tDR-0009 and tDR-7336 are mainly involved in the resistance of TNBC to doxorubicin; tRF-315 can reduce the sensitivity of PC cells to cisplatin by targeting GADD45A, thereby preventing cisplatin-induced apoptosis.

tsRNAs play an essential role in regulating the proliferation of tumor cells, which may contribute to tumor progression (Fig. 4A). tRF-1001 is generated by ELAC2 cleavage of pre-tRNA-SerTGA. The knockdown of tRF-1001 can impair proliferation, leading to a reduction in DNA synthesis and an accumulation in the G2 phase of the cell cycle.^47^ In 2015, Honda et al observed that the knockdown of three 5′-SHOT-RNAs (5′-SHOT-RNA^LysCUU^, 5′-SHOT-RNA^AspGUC^, and 5′-SHOT-RNA^HisGUG^) in androgen receptor-positive PC and estrogen receptor-positive BC cell lines inhibited cell proliferation.^136^ tRF5-Glu can bind to the 3′UTR of breast cancer anti-estrogen resistance 3 (BCAR3) mRNA to inhibit its expression, thereby hindering the proliferation of ovarian cancer cells.^137^ In addition, Farina et al identified four tsRNAs (ts-19, ts-29, ts-46, and ts-112), of which ts-112 is the only tsRNA affected by tumor suppressor RUNX1 and can enhance the proliferation of mammary epithelial cells.^138^ A recent study showed that transfection with tRF-Leu-CAG inhibitor decreased the proliferation of H1299 cells and increased the number of G0/G1 phase cells. Additionally, aurora kinase A (AURKA) expression was inhibited,^139^ suggesting that tRF-Leu-CAG may regulate the cell cycle by affecting AURKA activity. In the Epstein–Barr virus (EBV) immortalized B lymphocyte line (LCL) model, lactate (LA)-induced 5′tiRNA-His-GUG competitively binds to the chromatin regulator AGO2, resulting in a decrease in the association of AGO2 with miR-20a thereby leading to an increased expression of its target genes SFMBT1 and MAP3K7, and ultimately promoting the proliferation of B lymphoma cells.^140^

### Tumor metastasis

Upon separation from the primary tumor, these cells enter nearby blood vessels and lymphatic vessels, forming small cell nodules and continuing growth (Fig. 4B).^141^ Londin et al found that dysregulated tsRNAs in uveal melanoma (UVM) metastasis samples can regulate metastasis by affecting the activity of retrotransposons, and their regulatory mechanisms vary with the length of tsRNAs.^131^^,^^142^ Endothelial-mesenchymal transition (EMT) is critical in regulating migration.^143^ Under hypoxia, tRF-20-M0NK5Y93 can inhibit CRC cell migration and invasion by targeting EMT-associated Claudin-1.^144^ In addition, tRF-19-W4PU732S can promote the progression of BC cells by inhibiting the expression of ribosomal protein-L27A (RPL27A) and inducing EMT and cancer stem-like cells (CSC) phenotypes.^145^

The activation of oncogenic signaling pathways also improves the metastatic ability of tumor cells. For example, tRF/miR-1280 inhibits the Notch signaling pathway by targeting JAG2, ultimately inhibiting CRC metastasis.^87^ 5′-tiRNA^Val^ inhibits the migration and invasion of BC cells by targeting FZD3 to inhibit the Wnt/β-catenin signaling pathway.^146^ Nucleolin (NCL) is an RBP overexpressed in BC, and tRF3E derived from tRNA-Glu can form a complex with NCL, thereby disrupting the inhibitory effect of NCL on p53 mRNA translation, leading to increased p53 expression and thus inhibiting tumor growth.^147^

### Cell apoptosis

Apoptosis, also known as programmed cell death, is one of the major obstacles in the pathogenesis of cancer (Fig. 4C).^141^ Apoptosis is initiated by the release of cytochrome c (Cyt c) from the mitochondria into the cytoplasm, followed by the binding of Cyt c to the caspase activator Apaf-1, which allows Apaf-1 to assemble into the oligomeric apoptosome complex and trigger apoptosis.^148^ It has been reported that tRNAs binding to Cyt c can hinder the interaction of Cyt c with Apaf-1 and inhibit caspase-9 activation, eventually inhibiting apoptosis.^149^ As tsRNAs are derived from tRNAs, they are likely to possess similar functions as tRNAs, enabling cancer cells to resist apoptosis. Saikia et al demonstrated that during hyperosmotic stress, tiRNAs interact with Cyt c released from the ribonucleoprotein (Cyt c-RNP) complex in mouse embryonic fibroblasts (MEFs), preventing the formation of the apoptosome, suggesting that tiRNAs may also play a role in the apoptosis of tumor cells.^150^ Furthermore, Chen et al found that ALKBH3 can catalyze the demethylation of m^1^A and m^3^C in tRNAs, resulting in tRNAs being more sensitive to ANG cleavage to generate tsRNAs, which subsequently interact with Cyt c to prevent apoptosis of HeLa cells.^67^

It has been reported that although ANG-mediated tiRNAs inhibit global translation, they can selectively enhance the translation of anti-apoptotic genes.^20^ The translation of eukaryotic mRNAs is usually 5′-cap-dependent; under stressful conditions, this cap-dependent translation is turned off, and the organism uses alternative mechanisms to trigger translation. One of these translation pathways is initiated by the IRES. In this case, the 5′-end short RNA sequence of the gene can fold into a structure like the initial tRNA, mediating the binding of rRNAs and initiating translation.^18^^,^^20^ IRES-translated genes are commonly associated with anti-apoptosis, including Bcl-2,^151^ BAG-1,^152^ HiaP2,^153^ and c-Myb.^154^ However, tRNAs can also regulate apoptosis sensitivity, so it is unclear whether the two types of RNAs are antagonistic.

### Metabolic disorders

Cancer is characterized by abnormal energy metabolism (Fig. 4D). tRF^Lys−CTT−010^ can promote triple-negative breast cancer (TNBC) progression by targeting the glucose-6-phosphatase catalytic (G6PC) subunit to regulate lactate production and glycogen consumption.^155^ In addition, tsRNAs can also be involved in regulating lipid biosynthesis. For example, tsRNA-06018 can regulate adipogenic differentiation in human bone marrow mesenchymal stem cells (hMSCs) by targeting Stanniocalcin 2 (STC2) through the extracellular signal-regulated kinase 1/2 (ERK1/2) signaling pathway.^156^ In alcoholic fatty liver disease (AFLD), complement C3 can regulate its early severity. C3 activation products C3a and Asp increase the expression of Gly-tRFs by mediating the expression of CYP2E1, and then Gly-tRFs enhance adipogenesis and inhibit fatty acid β-oxidation by targeting the 3′UTR of sirtuin1 (SIRT1).^157^

### Therapeutic resistance

Given their properties and biological functions, tsRNAs may be associated with chemoresistance, one of the most important barriers to cancer treatment (Fig. 4E). Sun et al found that patients with high expression of tRF-30-JZOYJE22RR33 and tRF-27-ZDXPHO53KSN have worse efficacy after trastuzumab treatment than patients with low expression and are associated with shortened progression-free survival (PFS), suggesting that tRF-30-JZOYJE22RR33 and tRF-27-ZDXPHO53KSN may be intervention targets for trastuzumab-resistant BC treatment.^158^ In addition, when the SUM-1315 cell line is stimulated by hypoxia, tDR-0009, derived from tRNAGly-GCC-1-1, and tDR-7336, derived from tRNAGly-GCC-1-2, are significantly up-regulated. These two tsRNAs are mainly involved in the resistance of TNBC to doxorubicin by maintaining stem cell number and cell response to interleukin (IL)-6 and regulating the activation of STAT3 phosphorylation.^159^ Analogously, in PC, tRF-315 can reduce mitochondrial dysfunction and the sensitivity of PC cells to cisplatin by targeting the cell cycle-related gene GADD45A, thereby preventing cisplatin-induced apoptosis.^160^ As research on the mechanisms underlying therapeutic resistance in tumors continues, a new phase of clinical treatment with anticancer drugs may be ushered in.

## Clinical value of tsRNAs in cancers

### Potential for diagnosis and prognosis

tsRNAs are widely distributed *in vivo* and can be detected in tumor tissues and body fluids. Several nucleotide modifications, such as m5C and N-methylguanosine (m2G), can prevent ribonucleases from degrading tsRNAs.^161^ Furthermore, tissue-specific and time-specific tsRNAs can improve diagnostic efficacy and predict the progression of tumors.^12^^,^^162^ All these properties suggest that tsRNAs have the potential to become biofluid-based noninvasive tumor markers (Table 1).

**Table 1:** Clinical value of tsRNAs in cancers.

Source	Cancer type	tsRNAs name	tsRNAs type	Expression	Clinical significance	Reference
Serum/plasm/tissue	GC	tiRNA-5034-GluTTC-2	5′ tiRNA	Down	Diagnostic efficacy reaches its highest when tissue and plasma are combined	^17^
	GC	tRF-19-3L7L73JD	i-tRF	Down	Associated with tumor size and has diagnostic value for GC	^163^
	GC	tRF-33-P4R8YP9LON4VDP	5′ tiRNA	Down	Novel biomarkers and targets for GC therapy	^164^
	GC	tRF-5026a	tRF-5	Down	Has diagnostic potential and predictive value for overall survival	^186^
	CRC	5′-tRF-GlyGCC	tRF-5	Up	Positively correlated with CRC progression and metastasis	^165^
	CRC	tRF-phe-GAA-031, tRF-VAL-TCA-002		Up	Significantly correlated with metastasis and stages	^166^
	PCa	tRF-544	i-tRF	Down	Prognostic biomarkers for PCa recurrence	^167^
	PCa	tRF-315	i-tRF	Up		
	BC	5′-tiRNA^Val^	5′ tiRNA	Down	Significantly correlated with TNM stage and lymph node metastasis	^146^
	BC	tRF-Gly–CCC–001	tRF-1	Up	Biomarkers for the diagnosis of BC	^176^
	BC	tRF-Arg-CCT-017,tiRNA-Phe-GAA-003	tRF-1, 5′ tiRNA	Up	Serve as diagnostic and prognostic biomarkers of BC	
	BC	tRF-17-79MP9PP	tRF-5	Down	Diagnostic biomarkers and potential therapeutic targets for BC	^187^
	TNBC	tDR- 000620	5′ tiRNA	Down	Biomarkers and therapeutic targets for TNBC recurrence	^169^
	non-TNBC	tDR-7816	i-tRF	Down	Diagnosis of non-TNBC in potential patients	^188^
	LUAD	tRF-16-PSQP4PE, tRF-21-RK9P4P9L0	tRF-5	Up	Diagnosis efficacy reaches its highest when the three are combined	^170^
	LUAD	tRF-16-L85J3KE	i-tRF	Down		
	HCC	tRF-Gln-TTG-006	tRF-5	Up	Has high diagnostic value even in the early stages	^189^
	NSCLC	tRF-Leu-CAG	5′ tiRNA	Up	Significantly correlated with the stage progression of NSCLC	^139^
	PDAC	tsRNA-MetCAT-37, tsRNA-ValTAC-41, tsRNA-ThrTGT-23	tRF-3	Up	Diagnostic and prognostic biomarkers of PDAC	^171^
	CLL	i-tRF-GlyCCC	i-tRF	Down	Screen and prognostic monitor of CLL patients	^172^
	ccRCC	5′tRNA4-Val-AAC	5′ tiRNA	Down	Decreased significantly in advanced and hypofractionated ccRCC	^190^
Exosome	BC	tRF-Arg-CCT-017, tRF-Gly–CCC–001	tRF-1	Up	Has good diagnostic and prognostic potential	^176^
		tiRNA-Phe-GAA-003	5′ tiRNA			
	ESCC	tRNA-GlyGCC-5	tRF-5	Up	Preoperative biomarkers for adjuvant therapy	^177^
	HCC	tRNA-GluCTC-5	tRF-5	Up	Diagnostic biomarkers for HCC	^178^
	HCC	tRNA-ValTAC-3	tRF-3	Up		
	HCC	tRNA-GlyTCC-5, tRNA-ValAAC-5	5′ tiRNA	Up		

### tsRNAs in gastric cancer (GC)

tiRNA-5034-GluTTC-2 is significantly down-regulated in both GC tissues and plasma, and the area under the ROC curve (AUC) is 0.779 and 0.835, respectively. Additionally, the AUC increases to 0.915 when tissues and plasma are combined. Meanwhile, the overall survival rate of patients with low tiRNA-5034-GluTTC-2 expression is lower than that of patients with high expression.^17^ Similarly, the significantly down-regulated tRF-19-3L7L73JD and tRF-33-P4R8YP9LON4VDP in plasma can distinguish GC patients from healthy donors and have the potential to become GC diagnostic markers.^163^^,^^164^

### tsRNAs in colorectal cancer (CRC)

The diagnostic AUC of 5′-tRF-GlyGCC with high expression in CRC plasma is 0.882, and when combined with carcinoembryonic antigen (CEA) and carbohydrate antigen 199 (CA199), the AUC increases to 0.926.^165^ Chen et al induced EMT with TGF-β and found that tRF-phe-GAA-031 and tRF-VAL-TCA-002 are significantly up-regulated in CRC, which is correlated with distant metastasis and clinical stage. Subsequent overall survival (OS) analysis showed that highly expressed tRF-phe-GAA-031 and tRF-VAL-TCA-002 are associated with shorter survival in CRC patients.^166^

### tsRNAs in prostate cancer (PC)

Olvedy et al found that tRF-544 is significantly down-regulated in recurrent PC and more obvious in high-grade PC or PC with pathological stage 3. In contrast, tRF-315 expression is up-regulated in recurrent disease or high-grade PC. Notably, the higher the tRF-315/tRF-544 ratio, the worse the progression-free survival (PFS) and the shorter the time to disease recurrence.^167^ In addition, a recent study showed that increased levels of hormone-dependent 5′tiRNA are also associated with poor clinicopathological parameters and shorter recurrence time of PC.^168^

### tsRNAs in breast cancer (BC)

5′-tiRNA^Val^ differentiates BC patients from healthy controls with a sensitivity of 90.0% and specificity of 62.7% and low expression of 5′-tiRNA^Val^ is significantly associated with higher TNM stage and lymph node metastasis.^146^ In addition, low expression of tDR-000620 in TNBC and lymphatic metastasis are two independent poor predictors of recurrence-free survival.^169^

### tsRNAs in other cancers

tRF-16-L85J3KE in plasma can distinguish healthy donors from lung adenocarcinoma (LUAD) patients with an AUC of 0.99, higher than tRF-21-RK9P4P9L0 and tRF-16-PSQP4PE. When these three tsRNAs are combined, the accuracy is significantly better than a single indicator.^170^ Similarly, the combination of tsRNA-MetCAT-37, tsRNA-ValTAC-41, and tsRNA-ThrTGT-23, which are significantly up-regulated in the serum of patients with pancreatic ductal adenocarcinoma (PDAC), could assist in improving the diagnostic efficacy of CA19-9 in PDAC.^171^ The level of i-tRF-GlyCCC in peripheral blood mononuclear cells (PBMCs) of CLL patients is significantly decreased, and the OS of patients with positive expression of i-tRF-GlyCCC is decreased. Therefore, i-tRF-GlyCCC may be used for screening and prognostic monitoring of CLL.^172^

### tsRNAs in EVs and exosomes

tsRNAs have also been reported to be present in EVs and exosomes, which can protect molecules from degradation and regulate disease progression.^173^, ^174^, ^175^ The AUC of tRF-Arg-CCT-017, tRF-Gly–CCC–001, and tiRNA-Phe-GAA-003 in plasma exosomes of BC patients reaches 0.700 when combined. Meanwhile, patients with high expression of tRF-Arg-CCT-017 and tiRNA-Phe-GAA-003 are associated with poor disease-free survival rate (DFS) and OS.^176^ Li et al found that tRNA-GlyGCC-5 and an unnamed small RNA (created name: sRESE) are enriched in salivary exosomes of patients with esophageal squamous cell carcinoma (ESCC), a bi-sesncRNA signature (composing of tRNA-GlyGCC-5 and sRESE) can identify ESCC with high sensitivity and specificity, and the risk score for prognosis (RSP) is also better than small RNA alone in predicting ESCC prognosis.^177^ In addition, tsRNAs in exosomes of HCC patients also have diagnostic potential.^178^

### Targets for treatment

tsRNAs can not only become biomarkers for diagnosis and prognosis but also provide possibilities for targeted therapy. Because tsRNA changes in pathological states, different methods can be employed to intervene in disease progression, such as direct regulation of the abundance of tsRNAs and activation of ribonucleases to cleave maternal genes.^179^ Furthermore, interference strategies target miRNAs, such as locked nucleic acid (LNA)-modified oligonucleotides, anti-miRNAs oligonucleotides (AMOs), and “antagonists”, may also apply to tsRNAs.^37^ For example, Kim et al used LNA to inhibit oncogenic 3′tRF-LeuCAG from inducing apoptosis of HCC cells in a mouse model of HCC xenograft.^126^ Furthermore, tsRNAs mimics can be synthesized and introduced into cells or tissues to exert protective effects and slow down tumor progression for tsRNAs with tumor-suppressive effects.^37^

In general, oligonucleotide therapy is of most concern, which targets RNA at gene levels and is easy to design and synthesize.^180^^,^^181^ However, efficient delivery of oligonucleotides to target sites remains a challenge. Encapsulation of oligonucleotides with carriers such as cell-penetrating peptides, vitamin B12, or nanoparticles has been reported to improve the delivery efficiency of oligonucleotides to bacteria.^180^ Lipid nanoparticles (LNPs) have also been successfully applied to the COVID-19 mRNA vaccine.^182^ It is worth noting that endosomal escape is an important step in the oligonucleotide delivery process.^183^ Brown et al found that LNPs may be suitable for this step, but a reduced duration accompanies this.^105^^,^^184^ The endogenous tsRNAs usually contain modifications for stabilization, but synthetic tsRNA mimics do not contain these modifications and are relatively unstable.^185^ Therefore, the stability may be enhanced by wrapping oligonucleotides with vectors such as LNPs. To conclude, tsRNAs have the potential to optimize clinical treatment strategies, but there are still some unknown barriers to overcome.

## Conclusions and perspectives

tsRNAs have dual roles in promoting and suppressing cancer, and dysregulated tsRNAs have the potential to assist in monitoring patients and provide new possibilities for targeted therapy. However, the research on tsRNAs is still in its infancy. Firstly, the mechanism of tsRNAs is not yet in-depth. The study of RNAi has only recently expanded from the cytoplasm to include targets in the nucleus that may regulate transcription and splicing. Currently, there is a lack of studies that explore the application of nuclear functions of RISC to tsRNAs, and these studies need to be expanded. Furthermore, it has been reported that tsRNAs can prevent the target genes of miRNAs from being cleaved by interacting with miRNAs.^140^ Still, it has not been identified whether tsRNAs can form regulation networks with other ncRNAs to influence their biological functions. Finally, research on the diagnostic potential of tsRNAs is growing but has not yet been applied to clinical practice, so this will pose a challenge for future researchers.

In the near future, due to the deepening of sequencing technology and technological innovation, more tsRNAs will likely be identified and their intrinsic mechanisms will be studied in greater detail. It is anticipated that the emergence of tsRNAs will provide new opportunities for cancer diagnosis, prognosis, and treatment.

## Author contributions

Y.Z. drafted the first manuscript. X.G. and Y.L. checked and revised the manuscript. S.J. and Y.H. participated in the review design and helped modify the manuscript. All authors read and approved the final version of the manuscript.

## Conflict of interests

The authors declare that they have no competing interests.

## Funding

This project was supported by grants from the National Natural Science Foundation of China (No. 81871720, 82072363) and the Nantong Municipal Health Commission, Jiangsu, China (No. QA2020027).
